# Supplementary material for: Spatio-temporal associations between deforestation and malaria incidence in Lao PDR
Source: eLife. 2021 Mar 9;10:e56974. doi: 10.7554/eLife.56974 (PMC8024023; doi:10.7554/eLife.56974)
Supplement: Transparent reporting form [file elife-56974-transrepform.pdf]

Westbrook Centre, Milton Road
Cambridge CB4 1YG
UK
P 01223 855340
W elifesciences.org
T @elife

eLife's transparent reporting form

We encourage authors to provide detailed information *within their submission* to facilitate the interpretation and replication of experiments. Authors can upload supporting documentation to indicate the use of appropriate reporting guidelines for health-related research (see EQUATOR Network), life science research (see the BioSharing Information Resource), or the ARRIVE guidelines for reporting work involving animal research. Where applicable, authors should refer to any relevant reporting standards documents in this form.

If you have any questions, please consult our Journal Policies and/or contact us: editorial@elifesciences.org.

Sample-size estimation

- You should state whether an appropriate sample size was computed when the study was being designed
- You should state the statistical method of sample size computation and any required assumptions
- If no explicit power analysis was used, you should describe how you decided what sample (replicate) size (number) to use

Please outline where this information can be found within the submission (e.g., sections or figure legends), or explain why this information doesn't apply to your submission:

In this observational study, no sample-size estimation was needed nor performed.

Malaria incidence data was abstracted from the surveillance system in all the malaria registries available collected at health facilities in the study area over the study period. (See subsection "Malaria case data" of the "Materials and Methods" section)

Treatment-seeking data, used to adjust malaria incidence data, was abstracted from two cross-sectional surveys. The sample-size of those two cross-sectional surveys have been decided to answer research questions outside the scope of this study and their estimation do not pertain to this study. (See subsection "Study site and population" and subsection "Treatment-seeking data" of the "Materials and Methods" section)

eLife Sciences Publications, Ltd is a limited liability non-profit non-stock corporation incorporated in the State of Delaware, USA, with company number 5030732, and is registered in the UK with company number FC030576 and branch number BR015634 at the address Westbrook Centre, Milton Road Cambridge CB4 1YG, UK | March 2019

Replicates

- You should report how often each experiment was performed
- You should include a definition of biological versus technical replication
- The data obtained should be provided and sufficient information should be provided to indicate the number of independent biological and/or technical replicates
- If you encountered any outliers, you should describe how these were handled
- Criteria for exclusion/inclusion of data should be clearly stated
- High-throughput sequence data should be uploaded before submission, with a private link for reviewers provided (these are available from both GEO and ArrayExpress)

Please outline where this information can be found within the submission (e.g., sections or figure legends), or explain why this information doesn't apply to your submission:

No experiment was conducted in this study and no replicates was needed nor performed.

Instead, data from different sources were combined to assess how much of the variation in outcome Y (monthly village-level malaria incidence) could be explained by the variation in exposure X (percent area around villages that experienced deforestation). (See figure 8 in Methods section)

No outliers were encountered.

12% of malaria registries data were excluded from the analysis because of missing GPS coordinates (and therefore no exposure data). An additional 0.3% were excluded because of missing date (and therefore no outcome data). (See subsection "Geo-referencing" of the "Results" section)

Statistical reporting

- Statistical analysis methods should be described and justified
- Raw data should be presented in figures whenever informative to do so (typically when N per group is less than 10)
- For each experiment, you should identify the statistical tests used, exact values of N, definitions of center, methods of multiple test correction, and dispersion and precision measures (e.g., mean, median, SD, SEM, confidence intervals; and, for the major substantive results, a measure of effect size (e.g., Pearson's r, Cohen's d)
- Report exact p-values wherever possible alongside the summary statistics and 95% confidence intervals. These should be reported for all key questions and not only when the p-value is less than 0.05.

Please outline where this information can be found within the submission (e.g., sections or figure legends), or explain why this information doesn't apply to your submission:

Please see subsection “Statistical analysis” and subsection “Treatment-seeking data” of the “Materials and Methods” section for statistical analysis methods.

Please see subsection “Spatio-temporal analysis” of the “Results” section and supplemental material “S2: Travel times and treatment-seeking results” for statistical tests used.

Note that we purposely stayed away from any p-value. Not only can they be misleading, they do not provide any more information that the 95% confidence intervals, which have been reported for all estimates. In addition and importantly, our main results stress on the patterns of associations rather than on the statistical significance of any single association.

(For large datasets, or papers with a very large number of statistical tests, you may upload a single table file with tests, Ns, etc., with reference to sections in the manuscript.)

Group allocation

- Indicate how samples were allocated into experimental groups (in the case of clinical studies, please specify allocation to treatment method); if randomization was used, please also state if restricted randomization was applied
- Indicate if masking was used during group allocation, data collection and/or data analysis

Please outline where this information can be found within the submission (e.g., sections or figure legends), or explain why this information doesn't apply to your submission:

In this observational study, no group allocation was needed nor performed.

eLife Sciences Publications, Ltd is a limited liability non-profit non-stock corporation incorporated in the State of Delaware, USA, with company number 5030732, and is registered in the UK with company number FC030576 and branch number BR015634 at the address Westbrook Centre, Milton Road Cambridge CB4 1YG, UK | March 2019

Additional data files ("source data")

- We encourage you to upload relevant additional data files, such as numerical data that are represented as a graph in a figure, or as a summary table
- Where provided, these should be in the most useful format, and they can be uploaded as "Source data" files linked to a main figure or table
- Include model definition files including the full list of parameters used
- Include code used for data analysis (e.g., R, MatLab)
- Avoid stating that data files are "available upon request"

Please indicate the figures or tables for which source data files have been provided:

Source data (csv) and code (R) files for figures 2, 3, 4 and 5 and for tables 1, 2 and 3 have been uploaded in the submission.

In addition, all data generated or analyzed in this study have been uploaded in the submission as supporting files.
